# Transcatheter arterial infusion chemotherapy with cisplatin in combination with transcatheter arterial chemoembolization decreases intrahepatic distant recurrence of unresectable hepatocellular carcinoma

**DOI:** 10.1002/jgh3.12573

**Published:** 2021-05-18

**Authors:** Naoto Kawabe, Senju Hashimoto, Takuji Nakano, Kazunori Nakaoka, Aiko Fukui, Kentaro Yoshioka

**Affiliations:** ^1^ Department of Liver Biliary Tract and Pancreas Diseases, Fujita Health University School of Medicine Aichi Japan; ^2^ Faculty of Pharmacy Meijo University Aichi Japan

**Keywords:** cisplatin, hepatocellular carcinoma, transcatheter arterial chemoembolization, transcatheter arterial infusion chemotherapy

## Abstract

**Background and Aim:**

This study investigated the efficacy of transcatheter arterial infusion (TAI) chemotherapy with cisplatin combined with transcatheter arterial chemoembolization (TACE). The goal was to prevent intrahepatic distant recurrence (IDR) of hepatocellular carcinoma (HCC), compared with TACE alone, in patients with unresectable HCC.

**Methods:**

We conducted a historical cohort study, which involved 68 unresectable HCC patients. The study was performed on 44 and 24 consecutive patients who underwent TAI using cisplatin combined with TACE using epirubicin and TACE using epirubicin alone, respectively. We performed a propensity score analysis to identify the independent risk factors associated with IDR, and constructed propensity score‐adjusted survival curves.

**Results:**

After propensity score‐adjusting, the adjusted cumulative IDR rates at 1 and 3 years were 76.8 and 76.8% in TACE alone group, and 21.3 and 73.1% in TACE with TAI group, respectively. TACE alone group had a significantly higher IDR rate in comparison with TACE with TAI group (*P* = 0.0073). Combined with TAI was associated with preventing IDR after propensity score‐adjusting (hazard ratio [HR] 0.40, 95% confidence intervals [CI] 0.17–0.91, *P* = 0.028). Combined with TAI (HR 0.26, 95% CI 0.10–0.68, *P* = 0.0056) and Stage ≥III (HR 2.98, 95% CI 1.25–7.12, *P* = 0.014) were independent IDR predictors after adjusting for significant risk factors with propensity score.

**Conclusions:**

We demonstrated that cisplatin TAI accompanied with TACE decreased IDR compared with TACE alone. Our findings suggest that cisplatin TAI might contribute to a longer progression‐free period in unresectable HCC patients treated with TACE.

## Graphical abstract

Transcatheter arterial infusion (TAI) chemotherapy with cisplatin in combination with transcatheter arterial chemoembolization (TACE) decreases intrahepatic distant recurrence of unresectable hepatocellular carcinoma (HCC). These findings suggest that cisplatin TAI might contribute to a longer progression‐free period in unresectable HCC patients treated with TACE.

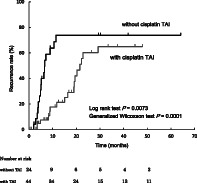

## Introduction

Recently, the number of deaths from hepatocellular carcinoma (HCC) is decreasing in Japan, but it remains that HCC is the sixth most common cancer and the third most common cause of cancer‐related death in the world.^1^ HCC's incidence is especially high in African and Asian countries, including Japan. This is because HCC has a higher incidence in patients with chronic hepatitis B or hepatitis C virus infection, which are highly epidemic in these countries.^2^

Currently, there is a wide range of HCC treatment methods, including liver transplantation, hepatectomy, radiofrequency ablation (RFA), percutaneous ethanol injection therapy (PEIT), transcatheter arterial chemoembolization (TACE), transcatheter arterial infusion (TAI) chemotherapy, molecular‐targeted chemotherapy, and radiotherapy.^3^ Such treatment methods can be used alone or combined. HCC's therapy should be selected with consideration for the liver's underlying clinical condition.

Recently HCC's treatment outcome has seen improvements. However, approximately 80% of patients who underwent curative resection develop an intrahepatic recurrence. The latter can be due to either intrahepatic metastasis from the primary lesion or multicentric carcinogenesis.^4^

Results of randomized controlled trials and meta‐analyses published after 2000 indicate that TACE provides a survival benefit for patients with unresectable or relapsed HCC.^5^, ^6^, ^7^, ^8^ However, there is a very high incidence of synchronous/asynchronous multicentric carcinogenesis and early‐stage intrahepatic metastasis. This is because HCC is predominantly associated with chronic hepatic disorders. The recurrence rate of stage I/II HCC 1 year and 2 years after RFA is 25%^9^ and 42%,^10^ respectively. Additionally, TACE has not been consistently useful for long‐term HCC treatment due to intrahepatic distant recurrence (IDR).

Few studies have reported on the effectiveness of additional chemotherapy combined with TACE to prevent IDR of HCC. Specifically, Ishikawa *et al*. compared the efficacy of TAI with cisplatin and TAI with carboplatin combined with curative treatment (RFA and/or TACE) to prevent IDR of the stage I/II HCC patients.^11^ They demonstrated that cisplatin obtained a significantly lower IDR rate compared with carboplatin. Based on these results, the authors concluded that cisplatin reduced IDR more effectively compared with carboplatin. In a retrospective study, Kim *et al*. evaluated the efficacy of arterial cisplatin infusion following TACE in patients with advanced HCC who had hepatic vein invasion and Child–Pugh class A.^12^ The authors concluded that significant prolonged survival was observed in patients accompanied with cisplatin TAI. However, to date no reports are available on the effect of TAI with cisplatin combined with TACE to prevent IDR of HCC compared with TACE alone in advanced HCC patients, including Child–Pugh class A and B, and stage III/IV with and without invasion in hepatic vein.

The aim of the present study was to investigate the efficacy of TAI with cisplatin combined with TACE for preventing IDR of HCC *vs* TACE alone in patients with unresectable HCC.

## Methods

### Patients

We performed a single‐center, retrospective cohort study. Specifically, we evaluated the efficacy of the first TACE combined with TAI using cisplatin (TACE with TAI) *vs* that of the first TACE without TAI (TACE alone) to prevent IDR of HCC. We considered including consecutive patients diagnosed as unresectable HCC and treated with TACE with epirubicin in this study. Patients were evaluated between April 2005 and March 2014 at Fujita Health University Hospital. In this period, the attending physicians decided the treatment method of TAI with cisplatin followed by TACE with epirubicin or TACE with epirubicin alone depending on the condition of each patient, mainly renal function considering the side effect of cisplatin. The liver function of the patients did not have significant difference between two groups.

Each patient had to meet the following criteria: previously untreated HCC or recurrent HCC after curative hepatectomy, RFA or PEIT, HCC for which neither hepatectomy nor local therapy (RFA or PEIT) was applicable, no extrahepatic metastasis, and an Eastern Cooperative Oncology Group performance status of 0–2. Tumor node metastasis (TNM) stage, which was determined as previously reported in studies for staging of HCC conducted by the Liver Cancer Study Group of Japan (LCSGJ),^13^ was used for evaluation of tumor progression.

Finally, 44 and 24 consecutive patients who underwent TAI with cisplatin followed by TACE with epirubicin and TACE with epirubicin alone were included in this study, respectively. Multivariate analysis identified the independent risk factors associated with IDR. There was selection bias between two groups, so a propensity score analysis was used for adjusting for the differences of baseline characteristics between the TACE+TAI group and TACE alone group as described in statistical analysis.

The ethics committee on human research of Fujita Health University approved this study. Each patient provided informed consent. The study protocol conformed to the guidelines of the 1996 revision of the Declaration of Helsinki.

### TACE and TAI

TACE was performed based on the following steps. A catheter was inserted into the feeding artery. There we monitored a densely stained tumor using angiography. An epirubicin‐lipiodol suspension (a mixture of 5 mL of lipiodol and 5 mL of a contrast medium containing 50 mg of epirubicin) was injected until the blood flow in the target artery stagnated according to the tumor size. The hepatic artery was embolized with porous gelatin particles from the feeding artery based on the tumor size and vascular diameter.

In the TACE+TAI group, a catheter was placed in proper hepatic artery. The cisplatin fine powder formulation (IA‐call; Nippon Kayaku, Tokyo, Japan) was solubilized in saline, at a concentration of 100 mg/70 mL, immediately prior to use. Cisplatin was administered for the whole liver from the proper hepatic artery with a total dose of 65 mg/m^2^. TACE with epirubicin was subsequently performed as mentioned above.

### Study endpoint and evaluation of therapeutic response

The present study defined IDR as the primary endpoint. According to Ishikawa *et al*.'s definition, IDR was determined as a new recurrent HCC occurring in another subsegment away from the area of previously treated HCC.^11^

The local recurrence was defined as the recurrence in the same subsegment with tumors underwent TACE. Because local recurrence before IDR may affect IDR, the overall local recurrence rates before IDR were evaluated in TACE alone group and in TACE+TAI group among patients with complete response.

Evaluation of therapeutic response of TACE was used enhanced CT results obtained at 1–2 months after TACE, in accordance with the modified RECIST guideline.^14^, ^15^ After the first evaluation, the follow‐up interval of CT scan is 1–3 months depending on the therapeutic response and the condition of the patients.

### Statistical analysis

Statistical analysis was conducted using the SAS 6.10 software (SAS Institute, Cary, NC, USA). Variables with a normal distribution were expressed as mean values ±SD, and asymmetrically distributed data were expressed as median and interquartile range (IQR). The differences between the two groups were evaluated by Student's *t*‐test or Mann–Whitney U test for continuous variables and the chi‐square test for categorical variables. Differences in the IDR rate between two groups were examined with the Kaplan–Meier method. Additionally, they were compared using a log‐rank test. Hazard ratios (HR) and 95% confidence intervals (CI) were calculated for each factor by a Cox proportional hazards analysis.

To adjust for the differences of baseline characteristics between two groups, a propensity score analysis was performed using multivariate logistic regression model including gender, age, and all baseline variables with significant differences between two groups. The score was subsequently incorporated into Cox proportional hazards model as a covariate. Furthermore, propensity score‐adjusted survival curves were also constructed. Finally, to determine independent predictors for the endpoints, we used Cox multivariate model that consisted of all covariates with *P* < 0.05 on the univariate analysis with propensity score. Specifically, the latter consisted of all covariates with *P* < 0.05 on the univariate analysis with propensity score. We considered differences with a *P* < 0.05 as statistically significant. The propensity score adjusting was performed because the number of patients was small in this study. The propensity score was used as a covariate not to decrease the number. The baseline characteristics after matching cannot be shown because matching with the propensity score was not performed in this study.

## Results

### Baseline characteristics

Baseline characteristics are described in Table 1. The prevalence of stage IV was significantly higher in TACE+TAI group than in TACE alone group (13.6 *vs* 0.0%, *P* = 0.023). Inversely, we observed that TACE+TAI group had lower prevalence of previous treatment (36.4 *vs* 70.8%, *P* = 0.0066), serum creatinine levels (0.75 ± 0.22 mg/dL *vs* 1.02 ± 0.41 mg/dL, *P* = 0.0009), and dose of EPI (20 ± 14 mg *vs* 34 ± 29 mg, *P* = 0.010) compared with TACE group. Other characteristics were comparable between two groups.

**Table 1:** Baseline characteristics

	All patients (*n* = 68)	TACE alone (*n* = 24)	TACE + TAI (*n* = 44)	*P* value
Gender (male/female)	51/17	20/4	31/13	0.24
Age (years)	71 ± 8	72 ± 6	70 ± 9	0.42
Etiology of liver disease (HBV/HCV/NBNC)	5/50/13	3/18/3	2/32/10	0.33
Previous treatment (surgical/local/non)	3/30/35	2/15/7	1/15/28	0.0066
Child‐Pugh class (A/B)	43/23	15/8	28/15	0.91
Child–Pugh score (5/6/7/8/9)	28/15/10/12/1	11/4/4/3/1	17/11/6/9/0	0.61
Stage (I/II/III/IVA)	7/22/33/6	5/5/14/0	2/17/19/6	0.023
Vp (−/+)	58/6	23/1	36/5	0.45
Number of tumor (1/2/3/>3)	15/6/4/42	6/3/1/14	9/3/3/28	0.73
Tumor diameter (mm)	31.1 ± 15.5	27.2 ± 14.5	33.2 ± 15.7	0.13
AFP (ng/mL)	48 (11–212)	52 (17–193)	44 (11–213)	0.52
DCP (mAU/mL)	89 (27–682)	52 (23–1072)	105 (30–682)	0.56
Creatinine (mg/dL)	0.85 ± 0.32	1.02 ± 0.41	0.75 ± 0.22	0.0009
Platelets (×10^4^/mm^3^)	9.9 ± 4.6	10.3 ± 4.7	9.8 ± 4.6	0.64
ALT (IU/mL)	58 ± 45	65 ± 63	54 ± 32	0.31
AST (IU/mL)	66 ± 38	65 ± 39	67 ± 37	0.81
Albumin (g/dL)	3.5 ± 0.5	3.6 ± 0.5	3.5 ± 0.5	0.19
Total bilirubin (mg/dL)	1.3 ± 0.7	1.1 ± 0.7	1.3 ± 0.6	0.19
Prothrombin time (%)	75.1 ± 11.5	73.3 ± 11.9	76.1 ± 11.5	0.34
Dose of epirubicin (mg)	25 ± 24	34 ± 29	20 ± 14	0.010
Dose of lipiodol (mL)	7.3 ± 4.3	7.1 ± 4.2	7.3 ± 4.4	0.85

*P* values represent the results of the comparison between TACE alone group and TACE with TAI group.

AFP, alpha‐fetoprotein; ALT, alanine aminotransferase; AST, aspartate aminotransferase; DCP, des‐gamma‐carboxy prothrombin; HBV, hepatitis B virus; HCV, hepatitis C virus; NBNC, both negative for HBV and HCV; TACE, transcatheter arterial chemoembolization; TAI, transcatheter arterial infusion; Vp, portal vein tumor thrombosis.

As we indicated in Table 1, Child–Pugh score and Child–Pugh classification of the patients did not have significant difference between two groups. There were no significant differences in The Barcelona classifications between two groups.

AFP was higher in the TACE group *vs* TAI/TACE group without significant difference, and the distribution of AFP between the two groups did not have significant difference between two groups.

### Treatment effects

Tumor responses 1–2 months after TACE are summarized in Table 2. Tumor responses, objective response rate (ORR), and disease control rate (DCR) 1–2 months after TACE were comparable between two groups (*P* = 0.89, *P* = 0.94, *P* = 0.94, respectively).

**Table 2:** Tumor responses 1–2 months after transcatheter arterial chemoembolization (TACE)

	TACE alone (*n* = 24)	TACE + TAI (*n* = 44)	*P* value
Response			0.89
CR	11 (45.8%)	19 (43.2%)	
PR	9 (37.5%)	18 (40.9%)	
SD	3 (12.5%)	5 (11.4%)	
PD	1 (4.2%)	2 (4.5%)	
ORR (CR + PR)	20 (83.3%)	37 (84.1%)	0.94
DCR (CR + PR + SD)	23 (95.8%)	42 (95.5%)	0.94

CR, complete response; DCR, disease control rate; ORR, objective response rate; PD, progressive disease; PR, partial response; SD, stable disease; TACE, transcatheter arterial chemoembolization.

Overall, local recurrence rates before IDR were 9.1% in TACE alone group, and 10.5% in TACE+TAI group among patients with complete response. Local recurrence rates before IDR had no significant differences between TACE+TAI group and TACE alone group.

### Intrahepatic distant recurrence rates

The cumulative IDR rates, by Kaplan–Meier analysis, at 1 and 3 years were 76.7 and 76.7% in TACE group, and 21.3 and 73.2% in TACE+TAI group, respectively. The TACE alone group had a significantly higher IDR rate compared with TACE+TAI group (*P* = 0.0071). Furthermore, median IDR‐free time was 6.5 months in TACE alone group and 21.4 months in TACE+TAI group (*P* < 0.0001).

### Predictors of intrahepatic distant recurrence

By means of the Cox univariate analysis, we identified the following characteristics as IDR predictors: combined with TAI (HR 0.39, 95% CI 0.19–0.79, *P* = 0.0093), Stage ≥III (HR 2.29, 95% CI 1.09–4.84, *P* = 0.029), creatinine levels (HR 1.11, 95% CI 1.01–1.23, *P* = 0.030), and dose of epirubicin (HR 1.02, 95% CI 1.00–1.03, *P* = 0.023) (Table 3).

**Table 3:** Predictors for intrahepatic distant recurrence by univariate Cox analysis

	Hazard ratio	95% confidence interval	*P* value
Combination with TAI	0.39	0.19–0.79	0.0093
Male	1.21	0.55–2.69	0.64
Age (per 1 year↑)	1.02	0.97–1.07	0.36
HCV versus HBV or NBNC	1.45	0.63–3.34	0.39
Previous treatment	0.98	0.49–1.94	0.95
Child–Pugh class (A versus B)	1.13	0.52–2.46	0.76
Stage ≥ III	2.29	1.09–4.84	0.029
Number of tumors >3	1.84	0.86–3.96	0.12
Tumor diameter (per 1 mm↑)	1.01	0.98–1.03	0.53
AFP > 48 ng/mL	1.02	0.48–2.16	0.96
DCP > 89 mAU/mL	1.24	0.59–2.61	0.56
Creatinine (per 0.1 mg/dL↑)	1.11	1.01–1.23	0.030
Platelets (per 1 10^4^/mm^3^↑)	1.01	0.95–1.09	0.69
ALT (per 1 IU/mL↑)	1.01	0.99–1.01	0.51
AST (per 1 IU/mL↑)	1.00	0.99–1.01	0.87
Albumin (per 1 g/dL↑)	0.83	0.42–1.62	0.57
Total bilirubin (per 1 mg/dL↑)	1.05	0.57–1.92	0.88
Prothrombin time (per 1%↑)	1.01	0.98–1.04	0.49
Dose of epirubicin (per 1 mg↑)	1.02	1.00–1.03	0.023
Dose of lipiodol (per 1 mL↑)	1.02	0.94–1.12	0.61

AFP, alpha‐fetoprotein; ALT, alanine aminotransferase; AST, aspartate aminotransferase; DCP, des‐gamma‐carboxy prothrombin; HBV, hepatitis B virus; HCV, hepatitis C virus; NBNC, both negative for HBV and HCV; TAI, transcatheter arterial infusion.

**Figure 1:**
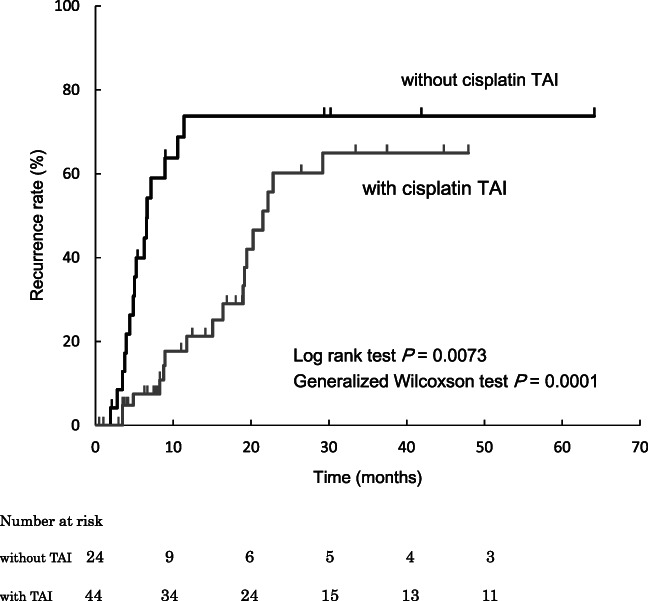
Cumulative intrahepatic distant recurrence rate. TAI, transcatheter arterial infusion.

Of note, combined with TAI was associated with preventing IDR even after propensity score‐adjusting (HR 0.40, 95% CI 0.17–0.91, *P* = 0.028, Table 3). Adjusted cumulative IDR rates at 1 and 3 years were 76.8 and 76.8% in TACE alone group, 21.3 and 73.1% in the TACE+TAI group, respectively (Fig. 1). The IDR rate was significantly lower in TACE+TAI group compared with TACE group (*P* = 0.0073). After adjusting for significant risk factors with propensity score including Stage and previous treatment, independent IDR predictors were combined with TAI (HR 0.26, 95% CI 0.10–0.68, *P* = 0.0056) and Stage ≥III (HR 2.98, 95% CI 1.25–7.12, *P* = 0.014) (Table 4).

**Table 4:** Predictors for intrahepatic distant recurrence by multivariate cox analysis

	Propensity score‐adjusted		Risk‐adjusted with propensity score	
	HR (95% CI)	*P* value	HR (95% CI)	*P* value
Combination with TAI	0.40 (0.17–0.91)	0.028	0.26 (0.10–0.68)	0.0056
Stage > III			2.98 (1.25–7.12)	0.014
Creatinine			1.13 (0.96–1.33)	0.13
Dose of epirubicin			1.01 (0.99–1.02)	0.26

CI, confidence interval; HR, hazard ratio; TAI, transcatheter arterial infusion.

There were not enough cases to compare the overall survival as an endpoint, because many enrolled patients' prognoses could not be confirmed since they were transferred from our hospital to other hospitals at the end stage of HCC. For the same reason, we could not show how many cases died from cancer after confirmation of recurrence.

### Adverse events

The adverse events related with the treatment were assessed according to the National Cancer Institute Common Terminology Criteria, version 4.0. We evaluated the adverse events as the maximum change in the grade within 1 month post‐treatment. Neither group developed adverse events of Grade 3 or 4.

We observed no differences among the two groups in terms of both adverse events caused by the treatments and laboratory tests post‐treatment. No specific adverse events were identified in two groups.

We observed temporary elevations of creatinine, total bilirubin, alanine aminotransferase (ALT), and aspartate aminotransferase (AST) levels, as well as temporary declines in the platelet count and albumin levels. These laboratory test values changed similarly in both groups. It should be noted that the tests returned to their pretreatment levels 2 months post‐treatment. Additionally, Child–Pugh scores changed similarly in both groups, and hepatic reserve capacity was temporarily reduced but returned to its pretreatment levels 2 months post‐treatment (Table 5).

**Table 5:** Changes in laboratory data before and 2 months after treatment

	TACE alone			TACE with TAI		
	Baseline	2 months after	*P* value	Baseline	2 months after	*P* value
Creatinine (mg/dL)	1.02 ± 0.41	1.00 ± 0.32	0.54	0.75 ± 0.22	0.76 ± 0.24	0.44
Platelets (×10^4^/mm^3^)	10.3 ± 4.7	8.3 ± 4.6	0.21	9.8 ± 4.6	11.7 ± 4.9	0.21
ALT (IU/mL)	65 ± 63	47 ± 42	0.17	54 ± 32	45 ± 30	0.18
AST (IU/mL)	65 ± 39	50 ± 38	0.16	67 ± 37	53 ± 32	0.19
Albumin (g/dL)	3.6 ± 0.5	3.5 ± 0.4	0.48	3.5 ± 0.5	3.4 ± 0.4	0.68
Total bilirubin (mg/dL)	1.1 ± 0.7	1.1 ± 0.6	0.65	1.3 ± 0.6	1.2 ± 0.5	0.38
Prothrombin time (%)	73.3 ± 11.9	75.8 ± 12.2	0.43	76.1 ± 11.5	77.5 ± 12.1	0.36
Child–Pugh Score	6.1 ± 1.7	6.2 ± 1.8	0.39	6.0 ± 1.4	6.2 ± 1.7	0.31

ALT, alanine aminotransferase; AST, aspartate aminotransferase; TACE, transcatheter arterial chemoembolization; TAI, transcatheter arterial infusion.

## Discussion

This is the first study demonstrating that TAI with cisplatin combined with TACE decreased IDR, compared with TACE alone, for unresectable HCC with and without hepatic vein invasion and including Child–Pugh class A and B. Our results suggest additional TAI with cisplatin may contribute to a longer progression‐free period for patients treated with TACE for unresectable HCC.

Nowadays, many systematic chemotherapies for HCC are being studied in clinical trials. These include multiple tyrosine kinase inhibitors and immune checkpoint inhibitors.^16^, ^17^, ^18^, ^19^ Unresectable HCC patients can now be administered sorafenib, regorafenib, lenvatinib, and ramucirumab in Japan.^20^, ^21^, ^22^, ^23^, ^24^ However, such systemic chemotherapeutic agents are limited to those patients with very good liver reserve function as only Child–Pugh class A. Specifically, many patients with HCC have underlying chronic liver diseases and declining liver function, so these systematic chemotherapies cannot be used for many HCC patients. Therefore, TACE still has very important position in the treatment strategy of unresectable HCC, especially for patients with poor liver reserve function as Child–Pugh class B.

Epirubicin,^25^, ^26^, ^27^ doxorubicin,^28^ mitomycin C,^29^ and cisplatin^30^ are commonly used in conventional TACE. However, their impacts on overall survival and response rate in conventional TACE are still unclear. Epirubicin is most frequently administered for the treatment of HCC combined with lipiodol, and it is approved in Japan for TACE.^31^ Although many randomized trials compared some treatment agents in combination with TACE for HCC, none of the agents have shown a survival benefit yet.^32^, ^33^, ^34^ The combination of the agents still does not influence clearly on the outcome of TACE.

Clear survival benefits of TACE for advanced HCC have been demonstrated in meta‐analyses.^5^, ^6^, ^7^, ^8^ As a consequence, TACE has been acknowledged as a palliative treatment for unresectable HCC. However, one of the main problems of TACE for HCC's treatment is the high recurrence rate, including local recurrence and IDR. While local recurrence could be controlled by the technical methods of TACE, controlling IDR through these methods is very difficult. This is due to intrahepatic metastasis and multicentric carcinogenesis of HCC.

TAI chemotherapy is frequently used to treat advanced HCC, which is generally associated with poor liver function. As opposed to systemic chemotherapy, TAI chemotherapy allows direct delivery of high doses of chemotherapeutic agents to the tumor site. Therefore, it reduces the systemic concentration of chemotherapeutic agents to a low level. As a result, patients may experience a lower incidence of adverse events.^11^ In an earlier study, Court *et al*. reported that, in cisplatin‐based chemotherapy, TAI enabled a greater drug accumulation within the tumor, compared to systemic chemotherapy.^12^

Cisplatin is consistent with a platinum complex compound, and its antitumor activity is divided into concentration‐dependent, fast‐acting, and slow‐acting groups. The absorbed percentage of cisplatin into HCC through first‐pass effects of TAI is reported as 48.4% (range, 34.2–55%).^35^

In a study performed by Ishikawa *et al*., they compared the effect of cisplatin TAI and carboplatin TAI combined with curative treatment (RFA and/or TACE). Their goal was to prevent IDR in HCC patients of Stage I/II.^11^ The authors demonstrated IDR rates were significantly lower in patients with cisplatin TAI than those of carboplatin TAI. They concluded that cisplatin had better effect than carboplatin for the prevention of IDR.

After their report, Kim *et al*. evaluated the effect of cisplatin TAI accompanied with TACE in patients with advanced HCC who had invasion in hepatic vein and Child–Pugh class A. The authors reported significant longer survival in patients who underwent cisplatin TAI than those who did not.^12^

However, there are currently no reports on the efficacy of TAI combined with TACE compared with TACE alone in patients with advanced HCC including Stage III/IV, with and without hepatic vein invasion, and including Child–Pugh class A and B. In the present study, we demonstrate that TAI with cisplatin combined with TACE decreased IDR compared with TACE alone in such condition of patients. These results prove the efficacy of TAI with cisplatin combined with TACE to prevent IDR of HCC under various conditions.

Ishikawa *et al* also reported that TAI using cisplatin in combination with TACE using miriplatin (Miripla; Dainippon Sumitomo Pharma, Osaka, Japan) improved survival compared with TAI using cisplatin accompanied with TACE using epirubicin in patients with HCC intermediate stage B of Barcelona Clinic Liver Cancer (BCLC) classification.^36^ They evaluated whether epirubicin or miriplatin best contributed to survival in TACE agents in combination with cisplatin TAI in primary HCC patients with BCLC‐B. However, they did not compare the efficacy or survival between TACE with TAI and TACE alone. The results of the present study are important because our data provide fundamental basic information and supplement their results.

Kamimura *et al*. demonstrated a randomized trial for evaluating the effect of TAI using combination of miriplatin and cisplatin *versus* miriplatin alone.^37^ They reported that the combination therapy of miriplatin and cisplatin had significantly better progression‐free survival and overall DCR. Recently, as miriplatin has become the standard drug in addition to anthracyclines in TACE in Japan,^38^, ^39^, ^40^ the results of the present study also provide useful basic information whether to add TAI using cisplatin to TACE using miriplatin in the future.

As to the adverse events caused by the treatments, no differences between TACE+TAI patients compared with TACE alone patients were observed. Additionally, no specific adverse events were identified in TACE+TAI patients compared with TACE alone patients. This is a retrospective study, which caused a selection bias between the two groups, particularly in renal function. Nevertheless, TAI with cisplatin in combination with TACE was able to be carried out safely just as TACE alone.

Several limitations can be identified in the present study. Primarily, this was a single‐center study with a limited number of patients. Secondly, this study was a retrospective cohort study, not a prospective randomized study. As a result, there might be confounding or residual selection bias in spite of propensity score adjustments. Thirdly, this study did not set the overall survival as an endpoint. This was because many enrolled patients' prognoses could not be confirmed since they were transferred from our hospital to other hospitals at the end stage of HCC. For the same reason, we could not show how many cases died from cancer after confirmation of recurrence. Therefore, our results should be considered in further investigations.

However, there are numerous patients with unresectable HCC treated with TACE since novel effective systemic chemotherapies have limited indications for patients who have good hepatic reserve function as Child–Pugh class A. Therefore, there is high need to further improve TACE's therapeutic effect to satisfy the unmet needs of patients with contraindications for effective systemic chemotherapies.

Our findings suggest that TAI with cisplatin might have a contribution to a longer progression‐free period for patients treated with TACE for unresectable HCC. Additionally, these results will contribute to future clinical practice and clinical trials.
